# Influence of *Fasciola hepatica* on Serum Biochemical Parameters and Vascular and Biliary System of Sheep Liver

**Published:** 2013

**Authors:** A Hodžić, A Zuko, R Avdić, A Alić, J Omeragić, A Jažić

**Affiliations:** 1 Department of Parasitology and Invasive Diseases, Veterinary Faculty, University of Sarajevo, Sarajevo, Bosnia and Herezegovina; 2 Department of Anatomy and Histology with Embriology, Veterinary Faculty, University of Sarajevo, Sarajevo, Bosnia and Herzegovina; 3 Department of Pathology, Veterinary Faculty, University of Sarajevo, Sarajevo, Bosnia and Herzegovina

**Keywords:** *Fasciola hepatica*, Sheep, Liver, Biochemical parameters, Corrosion cast

## Abstract

**Background:**

The aim of this study was to evaluate the functional capacity of the liver based on the activity of specific enzymes and bilirubin in serum and also to investigate the influence of mechanical and toxic effects of *Fasciola hepatica* on the structures of the blood vessels and biliary tract in the sheep liver.

**Methods:**

Blood samples and liver of 63 indigenous sheep of Pramenka breed, slaughtered in the period from March to December 2009 were used. Based on parasitological findings in the liver, all animals were divided into two groups: control (n = 34) and infected group (n = 29). For investigation and description of pathological changes in sheep liver, naturally infected with *F. hepatica*, corrosion cast technique was used.

**Results:**

Biochemical analysis of tested parameters showed a significant elevation (*P*≤0.05) of serum gamma-glutamyl transferase (GGT), total bilirubin (TBIL) and direct bilirubin (DBIL) in infected sheep group comparing with the control group. No significant differences were observed for activity of aspartate aminotranferase (AST) between groups. Vascular and biliary systems of the liver were found to be affected.

**Conclusion:**

Results of biochemical analysis are consistent with pathological findings and measuring of tested parameters could be used in early diagnosis of sheep fasciolosis and to test the effectiveness of anthelmintic therapy. Corrosion cast technique is very useful for investigation of pathological changes and neoangiogenesis of vascular and biliary system in sheep liver, caused by mechanical and toxic effects of *F. hepatica*.

## Introduction

Fasciolosis (Fascioliasis) is one of the most common parasitic disease in domestic animals, with a cosmopolitan distribution, particularly prevalent in countries with developed sheep production and very low socio-economic status, such as Bosnia and Herzegovina. It is caused by the hepatic trematode *Fasciola hepatica* (Linnaeus, 1758), which is mainly localized in the liver, bile ducts and gallblader of domestic and wild ruminants, especially in sheep. In animals, the subclinical and chronic disease usually results in decreased production of meat, milk and wool, secondary bacterial infections, fertility problems and great expenses with antihelmintics (1, 2).

The pathogenic effects of these flukes on the host organism begins with the ingestion of encysted metacercaria with vegetation or freshwater. After migration of juvenile forms through the hepatic parenchyma, flukes reside and graze on the mucosa of the bile ducts, which result in the massive tissue damage (3, 4). The lesions in the liver are only partially a result of mechanical action of liver fluke, because the injury of the liver can be induced by parasites excretory products, decomposed products of parasites, bile and hepatic tissue (5). Pathological changes, caused by mechanical and toxic effects of *F. hepatica*, affect the complex vascular and biliary system in the liver. Properly functioning of these two systems is the most important factor for preservation of normal liver functions.

Due to the complexity of vasular and biliary system examination, there are a number of different laboratory tests, which are based on biochemical analysis of serum parameters. These tests usually include the determination of serum transaminases (ALT, AST), which are the most sensitive indicators of hepatocellular injury. Alkaline phosphatase (ALP), gamma-glutamyl tranferase (GGT), serum proteins and bilirubin are also used to evaluate the degree of cholestasis and syntetic capacity of the liver (6–8).

Corrosion cast technique is usually used for anatomical investigations of three-dimensional vasculature and ductal architecture of some organs, and it has rarely been used for study of pathological changes in the liver caused by parasites or other pathogens (9).

In only a few studies authors have used a corrosion cast technique for investigation and description of pathological changes in liver in natural and experimental induced fasciolosis in sheep and cattle (9–12).

The aim of this study was to evaluate the functional capacity of the liver based on the activity of specific enzymes and bilirubin in serum and also to investigate the influence of mechanical and toxic effects of *F. hepatica* on the structures of the blood vessels and biliary tract in the sheep liver, using corrosion cast technique.

## Materials and Methods

### Animals

The study included serum and the liver of a total of 63 indigenous sheep of Pramenka breed, slaughtered in the period from March to December 2009. All sheep were from 1 to 3 years of age and body weight ranged 45 - 65 kg approximately. During the study, liver of the slaughtered animals was inspected for presence of *F. hepatica*. After that, all examined sheep, based on parasitological findings, were divided into two groups: control (n = 34) and infected group (n = 29).

### Blood samples and biochemical analysis

Before the animals were slaughtered, blood samples for biochemical analysis were collected from the external jugular vein into marked vacuum tubes and transported at +4 °C to the laboratories of the Veterinary Faculty in Sarajevo. The blood tubes were centrifuged at 3.000 rpm for 10 minutes for serum separation. Due to poor stability of enzymes and bilirubin in the serum, the samples were analyzed within 6 hours. Liver enzymatic activities of aspartate aminotransferase (AST), gamma-glutamyl transferase (GGT) and total bilirubin (TBIL) were measured according to standard procedures using automatic analyzer DIMENSION - Xpand ^PLUS^ (Dade Behring; Wien, Austria) with commercial kits (Flex^®^ reagent catridge - Siemens; Frimley, Camberley, UK). Direct bilirubin (DBIL) was determined by spectrofotometer BTS-330 (BioSystem; Barcelona, Spain), using modified Jandrassik/Gróf method (Human; Wiesbaden, Germany).

### Corrosion casts preparation

For this purpose a total of 63 liver were obtained from the slaughtered sheep. Thirty-four liver of sheep from control group were normal, without macromorphological changes, and twenty-nine were infected with *F. hepatica*. After dissection and fine preparation the blood vessels were rinsed with saline solution and heparin to remove remaining blood and the clots. The biliary tract was rinsed also. To prepare corrosion cast models, blood vessels and bile ducts of each liver, were injected with acryl resin (Interacryl cold - Interdent; Gornji Grad, Slovenia). Powder and solution of acrylat were mixed in a ratio of 1: 2 and then colored with different Biodur pastes. The portal vein, hepatic arteries, hepatic veins and bile ducts were stained green, red, blue and yellow, respectively. After hardening of resin at room temperature for 24 hours, the livers were macerated in 33% hydrochloric acid for 7-10 days. Later, corrosion casts were washed under tap water and air-dried (9, 13). In the second phase, the obtained casts were selected and studied, and the comparative differences between normal and infected liver casts were recorded.

### Statistical analysis

All statistical analyzes were performed using Minitab 15 software. Significant differences between infected and control group of animals were determined by Student's *t* test. Results are expressed as means±SD (standard deviation). Differences were considered as significant when *P*≤0.05.

## Results

### Biochemical analysis

The mean values of AST, GGT, TBIL i DBIL in serum of *F. hepatica* infected and control sheep and reference intervals are presented in Table 1. The results showed significantly higher activity of GGT and higher concentration of TBIL and DBIL in infected group than in the control group. No significant differences were observed for activity of AST between groups.

**Table 1:** Values of biochemical parameters in sheep serum of control and *Fasciola hepatica* infected group

Parameters	Control group (n = 34)	Infected group (n = 29)	Reference intervals (16)

	x±SD	x±SD	
AST (IU/l)	118,7±21,2	116,5±22,5	60 - 280
GGT (IU/l)	62,5±13,7	92,2±6,9***	20 - 52
TBIL(µmol/l)	4,7±0,8	5,9±0,8**	1,71 - 8,55
DBIL(µmol/l)	1,9±0,9	3,0±1,1***	0,0 - 4,61

** (*P*<0,01)

*** (*P*<0,001)

n = number of samples; x - means; SD - standard deviation

### Corrosion cast study

**Fig. 1:**
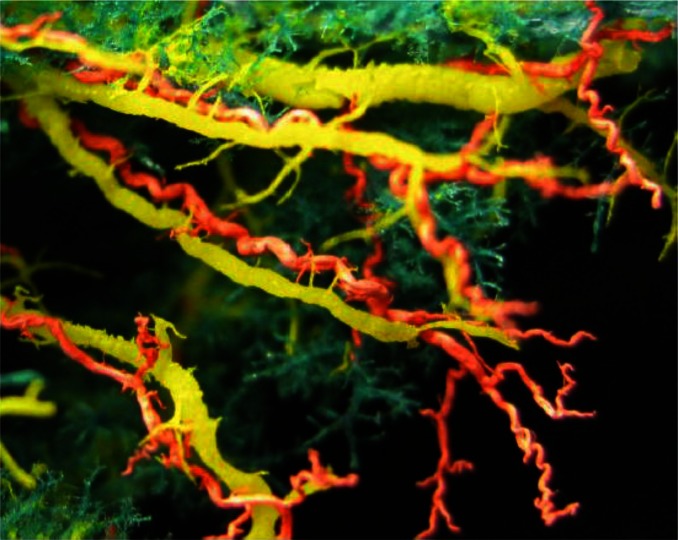
Corrosion cast showing dilated, rough and wringled bile ducts in left liver lobe, and reduction and lost of smaller branches (yellow) in a sheep infected with *F. hepatica*. Along to deformated bile duct are visible dilated and winding terminal branches of hepatic arteries (red)

Analysis of the corrosion casts, in the liver infected with *F. hepatica*, revealed dilated, rough and wrinkled bile ducts. Smaller ducts on the edges of intermediate and cranio-ventral segments of the left lobe were lost (Figs. 1 and 3).

**Fig. 2:**
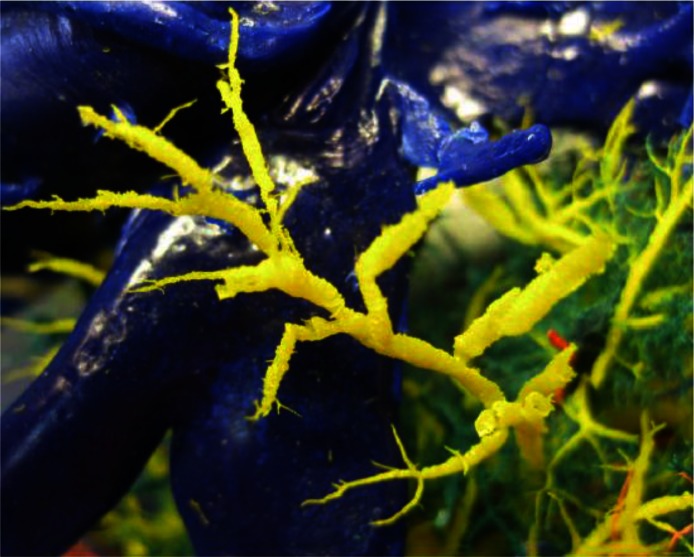
Newly formed bile ducts irregular in shape and positions (yellow)

In some cases, mostly in the caudate, quadrate and the ventral segment of the right lobe, newly formed bile ducts, not parallel with the terminal barnches of the portal vein and hepatic artery, were found. These ducts were divided in a number of smaller branches, whose diameter rapidly reduced toward the endings (Fig. 2).

**Fig. 3:**
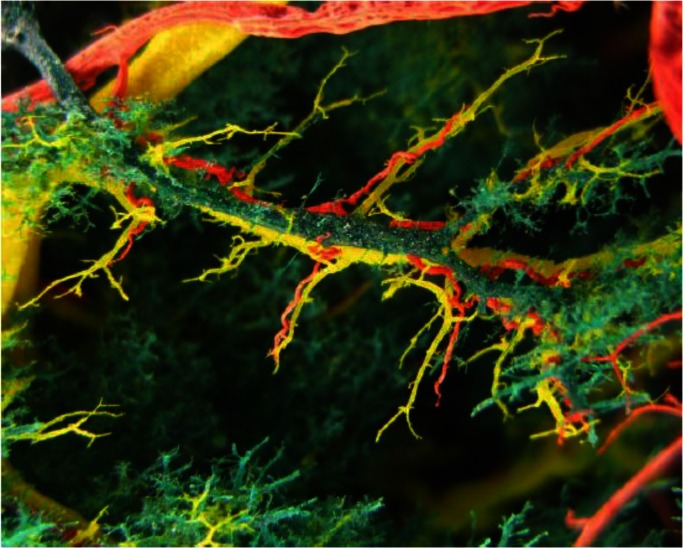
Cast of quadrate liver lobe showing narrowed terminal branches of portal vein and the reduction and loss of smaller portal (green) and hepatic (red) branches and bile ducts (yellow)

The secondary and terminal branches of the portal vein, along the dilated bile ducts, were narrowed and the smaller branches was reduced or lost. Similar changes, but less pronounced were established on the casts of the hepatic veins. These changes were mostly present in the caudate and quadrate liver lobes (Fig. 3).

**Fig. 4:**
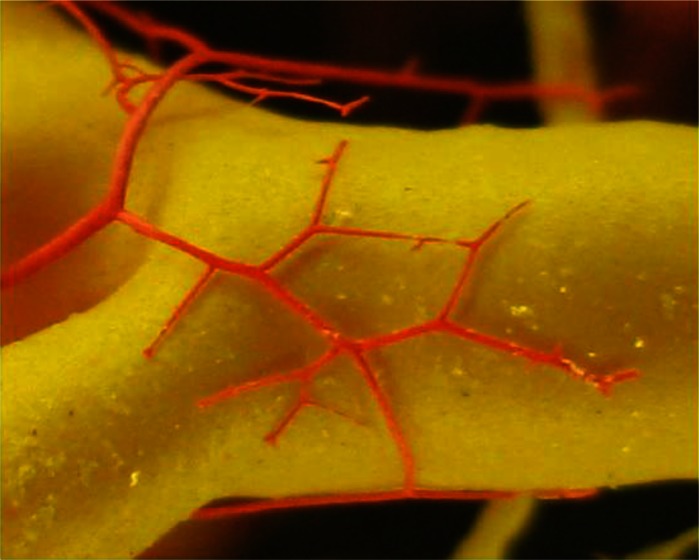
Hepatic arteries anastomoses (red) which surrounding the left hepatic duct (yellow)

Casts of the hepatic arteries, showed dilated and winding (serpentine shape) terminal branches (Figs. 1 and 3). In three livers, with the most pronounced macromorphological changes, we found vasculare anastomoses of hepatic arteries along the dilated hepatic ducts, surrounding them as a network (Fig. 4).

## Discussion

### Biochemical analysis

*Fasciola hepatica* causes the release of reactive oxygen species producing a damage of cell wall and hepatic tissue necrosis (14). These changes have influence on biochemical parameters in serum, and determination of specific liver enzymes is very valuable tool for diagnosis of hepato-biliary diseases. Physiologically, the normal levels of the enzymes in cells or serum is maintained by constant synthesis, simultaneous degradation, inactivation and elimination of enzymes (15). However, due to disruption of hepatocellular integrity, enzymes from damaged cells are released into the blood serum and their concentration increases above the physiological values.

In the present study, the obtained mean values of all tested biochemical parameters in the control sheep serum were within the reference ranges (16), except slightly higher value of GGT (Table 1). Increased activity of GGT in serum of clinically healthy animals indicates a moderate oxidative stress or occurs due to the intensification of metabolic processes, as a result of cell responses, especially the liver, on a negative energy balance (15).

Increased values of serum transaminases (ALT, AST) at the early stage of the infection could be related to the hepatocellular necrosis and degenerative changes produced by migration of juvenile flukes through the liver parenchyma (6, 14, 17, 18). Insignificantly higher levels of AST found in our study (Table 1), suggest lack of hepatocellular damage and probably indicate a chronic fasciolosis (biliary phase), regenerative changes in the parenchyma and the normalization of the liver function. According to Gonzalo-Orden et al. (19), activity of AST returns to normal values 11 weeks postinfection. Matanović et al. (20) detected significantly lower activity of AST in sheep serum from the infected herd, while Mert et al. (21) found significant elevation of AST in chronic fasciolosis, which is in contradiction with our findings.

Serum GGT values in infected sheep group were significantly higher than in the control group (Table 1). Some authors (17, 18, 20) reported that elevation in GGT levels was an indicator of chronic changes, cholestasis and epithelial damage in bile ducts caused by presence of adult flukes in biliary tract. However, Duff et al. (22) found that GGT activity increased in infected llamas due to hepatic toxicity and necrosis. Mert et al. (21) detected statistically significant elevation of GGT in sheep with chronic fasciolosis, aged 3-5 years. The obtained values in serum of control (22,2 ± 1,83 IU/l) and test group (44,4 ± 1,72 IU/ l) were significantly lower in comparison with ours, which can be explained that GGT activity physiologically decreases with age (1). Matanović et al. (20) have found approximately same values of GGT (93,24±24,81 IU/l) in organically farmed sheep naturally infected with *F. hepatica*.

In the present study, concentration of TBIL and DBIL were significantly higher compared to the control group (Table 1), but their values remained within the reference ranges (16). Our results were similar to that previously reported by Mekroud et al. (24) in 4-month old sheep experimentally infected with 150 and 300 metacercariae. They recorded a maximum concentration of bilirubin (9,57 µmol/l) in the week 15 postinfection. According to Taleb et al. (18), elevation of serum TBIL may be attributed to the increased production of bilirubin as a result of hemolytic toxins produced by liver fluke, while increase in the concentration of DBIL indicates bile ducts obstruction and intrahepatic cholestasis (6, 25). The biochemical changes in serum of *F. hepatica* infected sheep recorded in the present study were confirmed by corrosion cast study.

### Corrosion cast study

Changes observed on the casts of bile ducts, arterial and venous blood vessels of the sheep liver from infected group, completely correspond to changes described by Rushton and Murray (11, 12) and Shirai et al. (9) in naturally and experimental fasciolosis in sheep and cattle. Wrinkles and roughness of the cast of the bile ducts and loss of continuity (Figs. 1 and 3) occurs as a result of compression caused by inflammatory infiltrate, edema and proliferated connective tissue (4, 18). In the chronic stage of disease, these changes are the results of deposition of calcium salts on the damaged walls of the ducts (4, 5).

According to Rushton and Murray (11), duct dilation, especially at periphery of liver lobes, may occur as a result of larger aggregates of fluke eggs. Monolobular fibrosis with newly formed branched bile ducts have been observed in experimental fasciolosis in sheep, suggesting the regeneratory activity of liver parenchyma. Our results for bile ducts are in accordance with these findings. In this paper, corrosion casts of caudate, quadrate and ventral segment of right liver lobes show newly formed bile ducts that are not parallel with terminal branches of the portal vein and the hepatic artery (Fig. 2).

Observed narrowing of the secondary and terminal branches of portal vein along changed bile ducts is most probably a consequence of exudation and inflammation (edema), as well as, secondary proliferation of connective tissue in the chronic phase of the disease (Fig. 3). The same lesions caused morphologic alterations on the casts of the hepatic veins.

Malformations such as curved (serpentine) shapes, dilations, and loss of continuity on the casts of hepatic artery (Fig. 3), we believe are the consequence of aforementioned lesions. The reaction of arterial wall to injury caused by mechanical action of the parasitic migration has also contributed to their development. The migration of juvenile forms of the parasite damages the arterial and vein walls and leads to thrombosis and hyperplasia that result in dilation and curved (serpentine) shapes. The hyperplasia could also be induced by proliferation of the connective tissue in portal and interlobular spaces, which in turn explains the changes observed in chronic fasciolosis. The obstruction of vascular flow leads to neoangiogenesis and formation of anastomoses observed around bile ducts (Fig. 4). The development of new venules as a result of vascular regeneration has been observed by Preet and Prakash (26) in experimental cysticercosis in rats.

## Conclusion

Activities and concentration of AST, GGT, TBIL and DBIL in serum are reliable indicators of the stage of sheep fasciolosis and could be used in early diagnosis and to test the effectiveness of anthelmintic therapy. Corrosion cast technique is very useful for investigation of pathological changes and neoangiogenesis of vascular and biliary system in sheep liver, caused by mechanical and toxic effects of *F. hepatica*.
